# Rehabilitation exercise for treatment of vestibular disorder: A case study

**DOI:** 10.1100/tsw.2006.19

**Published:** 2006-02-28

**Authors:** Avraham Feazadeh, Eli Carmeli

**Affiliations:** Degani Institute, Institute of Physiotherapy, Hadera and Department of Physical Therapy, and Raziel Institute, Institute of Physiotherapy, Netanya, Israel, Sackler Faculty of Medicine, Stanley Steyer School of Health Professions, Tel Aviv University, Ramat Aviv 69978, Israel

**Keywords:** vertigo, dizziness, vestibular maneuver, Israel

## Abstract

Vertigo and dizziness are common symptoms in the general population. While the clinical picture is well known and widely described, there are different interpretations of Benign Paroxysmal Positional Vertigo. The purpose of this case report was to describe the treatment of a 56 year old woman with complains of positional vertigo for 35 consecutive years. She suffered from a sudden onset of rotatory, unilateral horizontal canal type benign paroxysmal positional vertigo (BPPV). The symtoms started a day after falling from a bus, where she injured her head. Otherwise her medical history was unremarkable. She was treated with an individualized home exercise program of eye movement exercises, Brandt/Daroff exercises, and general conditioning exercises (i.e., laying on the left side from sitting on the bed, while the head rotated 45 degrees to the right, waiting for about one minue; twice a day on gradual basis, not laying on the side all the way, but to use enough pillows to lay about at 60 degrees). Four weeks from the start of physical therapy, the patient was free of symptoms, even when her neck was in the extended position.

## INTRODUCTION

In Benign Paroxysmal Positional Vertigo (BPPV), dizziness is thought to be due to debris collected within a part of the inner ear. This debris can be thought of as “ear rocks”, although the formal name is “otoconia”. Ear rocks are small crystals of calcium carbonate derived from a structure in the ear called the “utricle” (see Fig. 1). While the saccule also contains otoconia, they are not able to migrate into the canal system. The utricle may have been damaged by head injury, infection, or other disorder of the inner ear, or may have degenerated due to advanced age. Normally, otoconia appear to have a slow turnover. They are probably dissolved naturally as well as actively reabsorbed by the “dark cells” of the labyrinth[1], which are found adjacent to the utricle and the crista, although this idea is not accepted by other investigators[2].

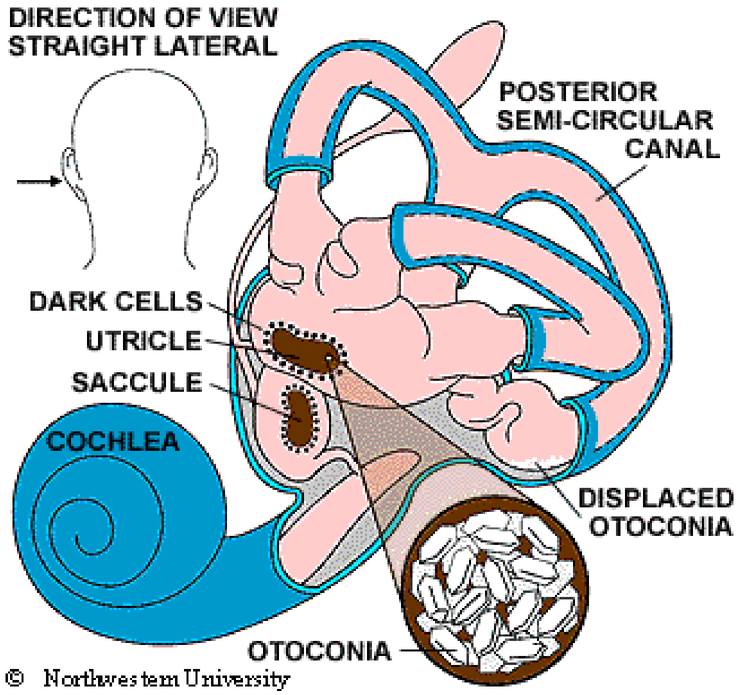

**FIGURE 1**. Structure of the inner ear (courtesy of Northwestern University).

BPPV is a common cause of dizziness, about 20% of all dizziness is due to BPPV. The older you are, the more likely that your dizziness is due to BPPV, as about 50% of all dizziness in older people is due to BPPV, which seems part of human development. In a recent study, 9% of urban elders were found to have undiagnosed BPPV[3]. The symptoms of BPPV include dizziness or vertigo, lightheadedness, imbalance, and nausea. Activities that bring on symptoms will vary among different people, but symptoms are almost always precipitated by a change of position of the head with respect to gravity. Getting out of bed and rolling over in bed are common “problem” motions. Because people with BPPV often feel dizzy and unsteady when they tip their heads back to look up, sometimes BPPV is called “top shelf vertigo”. Women with BPPV may find that the use of shampoo bowls in beauty parlors can bring on symptoms. An intermittent pattern is common, where BPPV may be present for a few weeks, then stop, then come back again.

The most common cause of BPPV in people under age 50 is head injury, but there is also an association with migraine[4]. In older people, the most common cause is degeneration of the vestibular system of the inner ear. BPPV becomes much more common with advancing age[5]. In half of all cases, BPPV is called “idiopathic”, which means that it occurs for no known reason. Virus affecting the ear such as those causing vestibular neuritis, minor strokes such as those involving anterior inferior cerebellar artery (AICA) syndrome, and Meniere’s disease are significant, but unusual, causes.

## MANAGEMENT OF VESTIBULAR DISORDERS

BPPV is not a disease, but rather the outcome of many pathological or physiological processes. Thus, vestibular disorders that manifest with vertigo usually have a good prognosis. Most forms of vertigo have a benign cause and are characterized by spontaneous recovery of vestibular function or central compensation of a peripheral or central vestibular tone imbalance. Vertigo can be effectively relieved by pharmacological treatment, physical therapy, surgery, or psychotherapy. Occasionally, BPPV will follow after surgery, where the cause is felt to be a combination of a prolonged period of supine positioning, or ear trauma, when the surgery is to the inner ear[6]. There is no common treatment and vestibular suppressants provide only symptomatic relief. A specific therapeutic approach can include causative, symptomatic, or preventive approaches[7].

## CASE REPORT

This case of vertigo with a symptom continuation for 35 years is uncommon. A 56-year-old female presented to the Physiotherapy Institute. She was from middle socioeconomic class, a housewife with complaints of vertigo for about 35 years, which occurred following laying on the left side or on the back for about 1 min duration. Due to discomfort of vertigo, she had not been laying on her left side or the back for that long a period of time. She claimed the symptoms started a day after falling from a bus and injuring her head.

Past medical history was unremarkable. She had received ***betahistine hydrochloride 16 mg*** a histamine analogue (Agiserc, Agis) 3 months before for a total of 1 month without improvement in her symptoms.

On examination, the patient developed strong vertigo (8/10 Visual Analogue Scale) while laying on her left side that continued for about 1 min. CT scan of brain and cervical spine were normal. Neurological testing and Bera test were normal. Hallpik-dix maneuver was not performed due to painful neck extension and patient fear[8]. Laying on the left side produced mild, geotropic, rotational nystagmus. Vor test was positive. Romberg test was unremarkable. Saccade and Pursuit test were negative.

Course of treatments: Patient was given left-side laying exercises, which were a modification of the Brandt and Daroff maneuver[9] as follows: Laying the patient on the left side from sitting on the bed, while head rotated 45 degrees to the right, waiting for about 1 min or until the vertigo disappeared and then sitting again. Patient was instructed to perform left-side laying exercises at home, twice a day; however, on a gradual basis, not laying on the side all the way, but to use enough pillows to lay at about a 60-degree incline. The number of pillows was to be reduced every day, so that over 5 days, the patient would be able to lay on her side.

Follow-up: Re-examined 1 week later and treatment regimen continued; 1 month later, no sign and symptoms were found and patient discharged.

## CONCLUSION

Brandt and Daroff maneuver[9] to the affected side, combined with home exercise, is an effective treatment option for a chronic, unilateral, horizontal, canal BPPV. These results support two possible mechanisms of BPPV: displaced otoconia and a neural mechanism affecting interpretation of semicircular canal signals.

## BIOSKETCHES

**Avraham Feazadeh**, **BPT,** Degani Institute, Institute of Physiotherapy, Hadera, Israel and Raziel Institute, Institute of Physiotherapy, Netanya, Israel Tel:+972-523747636; E-mail: AVRAHAM65@012.NET.IL

**Eli Carmeli, BPT, PhD**, is currently a Senior Lecturer of Gerontology and Anatomy and the chairman of the Department of Physical Therapy, Stanley Steyer School of Health Professions, Sackler Faculty of Medicine, Tel Aviv University. He is also the Editor-in-Chief of the Israeli Journal of the Physiotherapy Society. Eli received his BPT degree from Sackler Faculty of Medicine, Tel Aviv University and his PhD degree from the Rappaport Faculty of Medicine at the Technion in Haifa. Postdoctoral research was conducted at the University of Florida in Gainesville. His research interests are investigating the aging process both on the cellular and clinical level. E-mail: elie@post.tau.ac.il Website: http://www2.tau.ac.il/Person/medicine/HealthSchool/researcher.asp?id=agfhfiffl
